# Abrupt stop of deep water turnover with lake warming: Drastic consequences for algal primary producers

**DOI:** 10.1038/s41598-017-13159-9

**Published:** 2017-10-23

**Authors:** Yana Yankova, Stefan Neuenschwander, Oliver Köster, Thomas Posch

**Affiliations:** 1 Limnological Station, Department of Plant and Microbial Biology, University of Zurich, Seestrasse 187, CH-8802 Kilchberg, Switzerland; 2 Zurich Water Supply, Hardhof 9, CH-8021 Zurich, Switzerland

## Abstract

After strong fertilization in the 20^th^ century, many deep lakes in Central Europe are again nutrient poor due to long-lasting restoration (re-oligotrophication). In line with reduced phosphorus and nitrogen loadings, total organismic productivity decreased and lakes have now historically low nutrient and biomass concentrations. This caused speculations that restoration was overdone and intended fertilizations are needed to ensure ecological functionality. Here we show that recent re-oligotrophication processes indeed accelerated, however caused by lake warming. Rising air temperatures strengthen thermal stabilization of water columns which prevents thorough turnover (holomixis). Reduced mixis impedes down-welling of oxygen rich epilimnetic (surface) and up-welling of phosphorus and nitrogen rich hypolimnetic (deep) water. However, nutrient inputs are essential for algal spring blooms acting as boost for annual food web successions. We show that repeated lack (since 1977) and complete stop (since 2013) of holomixis caused drastic epilimnetic phosphorus depletions and an absence of phytoplankton spring blooms in Lake Zurich (Switzerland). By simulating holomixis in experiments, we could induce significant vernal algal blooms, confirming that there would be sufficient hypolimnetic phosphorus which presently accumulates due to reduced export. Thus, intended fertilizations are highly questionable, as hypolimnetic nutrients will become available during future natural or artificial turnovers.

## Introduction

Global warming changes physical and chemical properties of lakes and catchments^1^, as well as their biota^2^. Inland surface waters are immediately affected by warming due to the strong correlation between air and surface water temperatures^2^ and the impact of increased solar radiation^3^. The documented average warming per annum is mainly attributed to striking temperature increases during cold seasons (winter) across the Northern Hemisphere^4,5^. However, in a recent study for a pre-alpine region, the strongest warming was observed during spring periods^6^. This causes an earlier onset and prolongation of thermal stabilization in even deep large lakes. Thus, chances of complete water turnover (holomixis), happening once or twice a year in the temperate zone, become smaller, owing to temperature-dependent density differences between warmer surface (epilimnion) and cold deep water layers (hypolimnion).

Depending on the morphometry and productivity of thermally stratified lakes, hypolimnetic oxygen is gradually depleted due to microbial respiration. Anoxia leads to a release of orthophosphate (PO_4_-P) otherwise bound in sediments at aerobic conditions. Thus vernal holomixis is not only fundamental for the replenishment of deep layers with oxygen but also for the transfer of nutrients from the hypo- to the epilimnion^7–9^. Nutrients in combination with higher irradiance, rising water temperature and reduced turbulence^10^ enable photoautotrophic organisms (algae) to develop massive populations, so called spring blooms^11^. This first boost in primary production is the basis for organisms’ successions within food webs^12,13^. Algal spring blooms are followed by high abundances of herbivores (e.g. protists^13^ and metazooplankton^14^) causing the breakdown of phototrophs, which is mirrored by high water transparency (the ‘clear water phase’). These reoccurring annual dynamics in temperate lakes seemed to be highly predictable and were described in detail by the PEG (Plankton Ecology Group) model and adaptations of this conceptual framework^12,14,15^.

Here we show that the typical annual successions within lake food webs may be at least partly lost in the absence of deep mixing. The phototrophic spring bloom community, commonly formed by diatoms and cryptophytes in temperate lakes^15^, is cut off from essential nutrients such as PO_4_-P and nitrogen (mainly nitrate NO_3_-N).This reduction in primary production will affect the entire food web as algae are the major source of substrates for bacteria and of food for consumers^13^. Ultimately, this phenomenon may propagate up to the level of top predators, causing drastic decreases in fish stocks^16^.

## Results and Discussion

**Figure 1:**
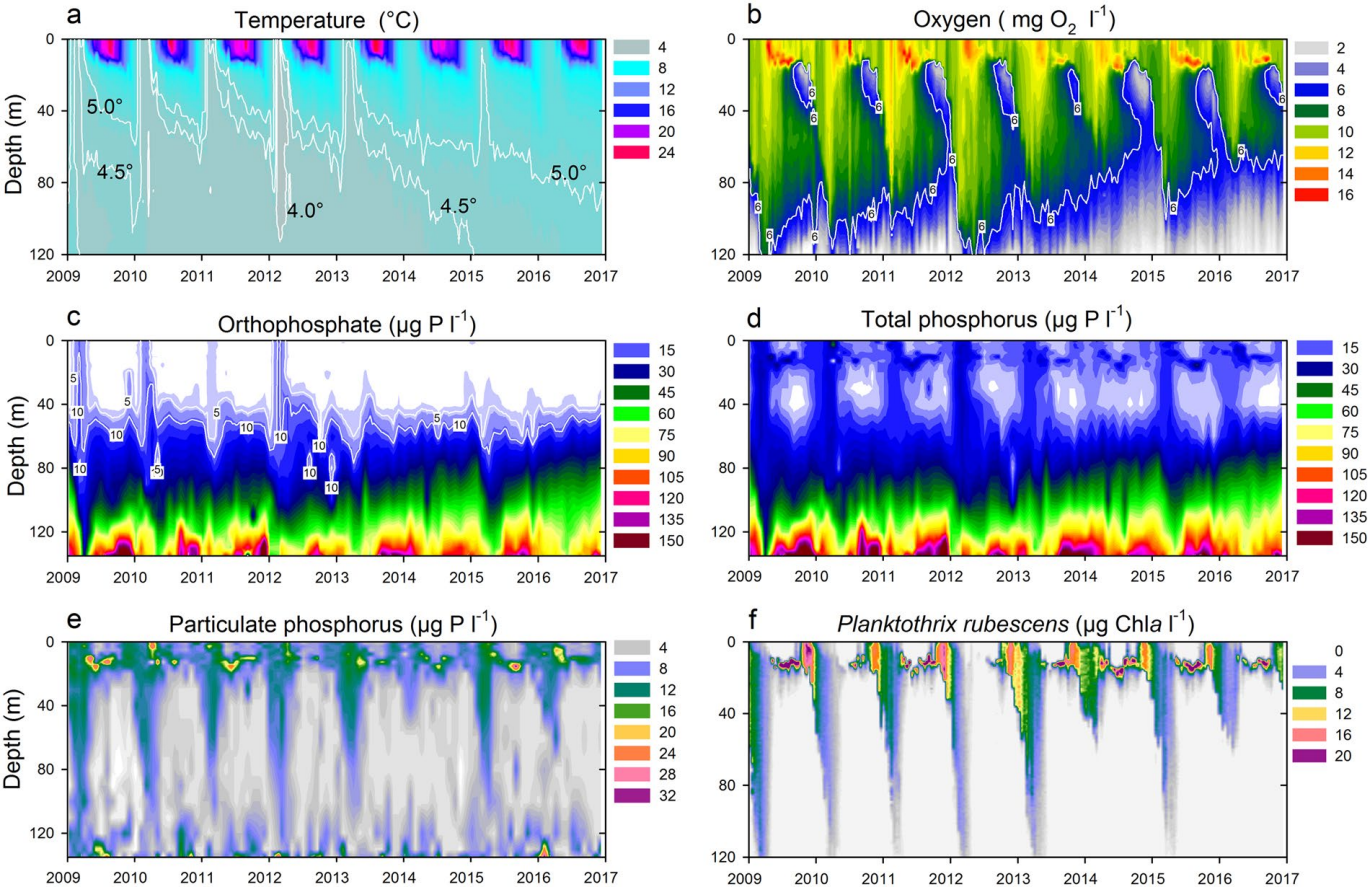
Recent trends (2009 to 2016) in warming, concentrations of oxygen and phosphorus, and cyanobacterial biomass in Lake Zurich (Switzerland). (**a**) Water temperature and two white isolines showing 4.5 °C and 5.0 °C, respectively. (**b**) Dissolved oxygen concentration and the 6 mg O_2_ l^−1^ isoline which is a proxy for the depth of maximal water turnover during spring. Note the metalimnetic oxygen minima developing each autumn expanding between 15–40 m water depths. Concentrations of dissolved orthophosphate (**c**), total phosphorus (**d**) and particulate (i.e., mostly organismic bound) phosphorus (**e**). (**f**) Total biomass (chlorophyll *a* concentration) of the most dominant primary producer in Lake Zurich, the cyanobacterium *Planktothrix rubescens*. Data based on biweekly profiles (*n* = 192) of parameters measured in 1 m depth intervals (0 to 120 m depth) in **a,b,f**. Data based on monthly profiles (*n* = 96) of 17 sampling depths (*n* = 1,632) in **c**,**d**,**e**.

Our study is based on a long-term monitoring of Lake Zurich (maximal depth: 136 m; Switzerland) which can be considered as a model ecosystem for temperate European lakes^3,17–19^. A strong relation between vernal epilimnetic PO_4_-P deficits and mixis depth became first obvious from a biweekly monitoring conducted since 2009. The overall warming of Lake Zurich in the last four decades has been documented^6,18,19^ and this trend continued during the last 8 years. Since 2013, warming steadily propagated in deeper water layers, and temperature increased to 5 °C even in 90 m depth (Fig. 1a). This extensive warming impeded vernal holomixis for four consecutive years (2013–2016). Lake Zurich is classified as warm monomictic, and the intensity of spring water turnover depends on isothermal conditions in combination with strong wind actions (velocities of >20 km h^−1^ are essential for deep water circulation)^20^. On the basis of long-term data sets several authors have shown that hypolimnetic oxygen concentrations and their increase during vernal circulation adequately reflect the intensity and depth of water turnover in Lake Zurich^21–23^. G.C. Örn (1980)^21^ evaluated hypolimnetic oxygen concentrations for the years 1937–1975 and defined a vernal value of ≥6 mg O_2_ l^−1^ as simple but valuable proxy for mixing depth (i.e. the water body which had O_2_ concentrations ≥6 mg O_2_ l^−1^ seemed to be affected by mixis). The validity of this proxy to describe mixis intensity^18^ was also shown for the continuation of the long-term dataset between years 1972–2011. Even if holomixis takes place, there is always a strong oxygen depletion starting from spring on in Lake Zurich. Oxygen depletion propagates from the ground upwards throughout the hypolimnion (Fig. 1b). Usually minimal oxygen concentration are reached in December, with an anaerobic zone above ground. Thus, vernal mixis in Lake Zurich always leads to an annually recurrent oxygen replenishment of deeper water strata (however, the spatial extension and quantity of replenishment depends on mixis intensity).

Here we show that the isoline referring to 6 mg O_2_ l^−1^ indicates a reduction of vernal mixis from 120 m in 2012 to only 69 m in 2016 (Fig. 1b and Supplementary Fig. S1). In parallel, the last large import of PO_4_-P from the hypo- to the epilimnion was observed in 2012 (Fig. 1c). Since then, concentrations in the water column down to 40 m were close to the limit of detection (<1 µg l^−1^) throughout the years. However, levels of biologically available PO_4_-P were still high (>150 µg l^−1^) in layers near to the sediment in late winter (Fig. 1c). Notably, concentrations of total phosphorus (TP = roughly the sum of dissolved PO_4_-P and particulate phosphorus) did not mirror the lack of holomixis at all (Fig. 1d). Most of TP in surface layers of lakes is made up by organismic bound phosphorus (particulate fraction, Fig. 1e), which in case of Lake Zurich highly corresponds to the dominant primary producer, the harmful cyanobacterium *Planktothrix rubescens* (Fig. 1f). This filamentous cyanobacterium regulates its buoyancy by gas vesicles within cells. Gas vesicles have to withstand the combination of cell turgor pressure and the hydrostatic pressure, which increases linearly with depth when filaments are entrained into the hypolimnion during vernal water turnover events. Genetic markers for their pressure resistance have been identified and in *P. rubescens* populations in Lake Zurich the dominant genotype was GV3, the strongest described variant. These vesicles have a critical collapse pressure of 1.17 MPa, thus filaments could retain buoyancy^24^ down to depths of 99 m. Hence, holomixis events result in the destruction^18,25^ of large fractions of cyanobacterial populations during winter/early spring (Supplementary Fig. S1). Thus, incomplete water turnovers allowed for high survival ratios, causing their permanent presence since autumn 2012 (Fig. 1f). This may have severe consequences for the entire food web as *P. rubescens* stores various toxic secondary metabolites making this harmful cyanobacterium an adverse food item for most consumers^26^. Meanwhile the vernal population dynamic of *P. rubescens*, i.e. the entrainment of filaments into deeper water layers, can be regarded as an additional proxy to determine the depth of water turnover in spring (Supplementary Fig. S1).

**Figure 2:**
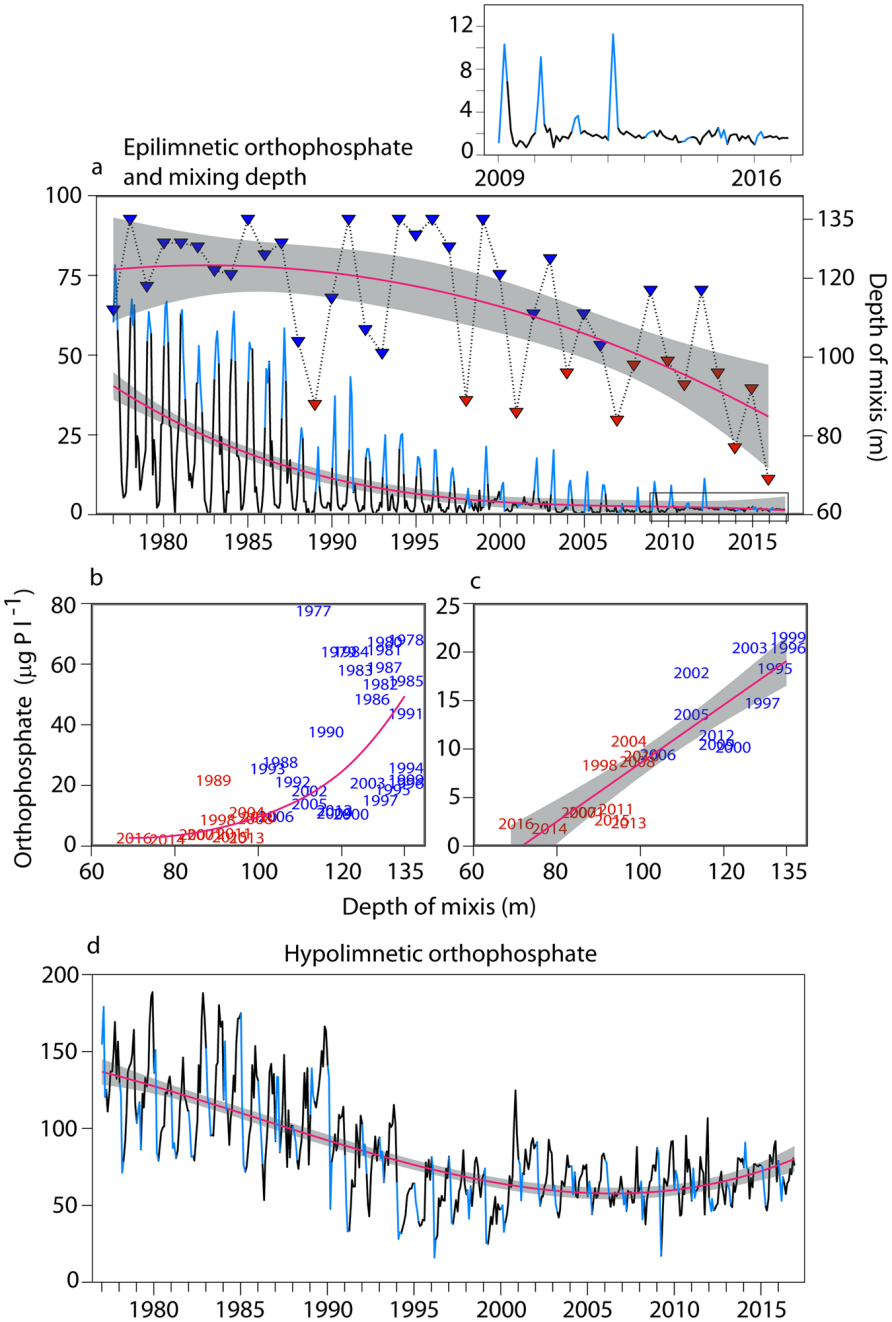
Long-term trends (1977 to 2016) of mixing depth and orthophosphate concentrations. (**a**) Dotted line and triangles: mean water turnover depth (*n* = 40) with color code for years with mixing depth <100 m (red) and ≥100 m (blue), and regression line (*f* = *y*_*0*_ + a*x + b*x ^2^ *, r* ^2^ = *0.4*2*, p* = *0.08*). Solid line: epilimnetic orthophosphate concentrations (*n* = 480) with highlighted periods of water turnover (January to April, light blue) and regression line (*f* = *y*_*0*_ + a*x + b*x ^*2*^ + c*x ^3^ *, r* ^2^ = *0.43, p* < *0.05*). The period 2009 to 2016 is enlarged to show that epilimnetic phosphorus enrichment stopped since the year 2013. (**b**) Relationship between mean mixing depth and epilimnetic orthophosphate for the years 1977 to 2016 (*f* = *y*_0_*e^b*x^, *r* ^2^ = *0.60*, *p* < *0.001*), and (**c**) 1995 to 2016 (*f* = b*x + *a*, *r* ^2^ = *0.79*, *p* < *0.001*) with colors as in (**a**). (**d**) Hypolimnetic orthophosphate concentrations (solid line, *n* = 480) with highlighted periods of water turnover (January to April, light blue) and regression line (*f* = *y*_*0*_ + a*x + b*x ^2^ + c*x ^3^, *r* ^2^ = *0.53, p* = < *0.01*). Grey shaded areas in (**a,c,d**) show confidence intervals.

In a next step the long-term data-set starting from 1977 (Fig. 2) was evaluated i) to test if multiannual series of incomplete water turnovers happened already before 2013, and ii) to define the level of ‘expected’ epilimnetic PO_4_-P enrichment during spring mixis. This data-set is based on monthly sampling of 19 distinct depths (along 0–136 m) resulting in a total of >9,100 values per biological and physico-chemical parameter.

While water turnover expanded deeper than 90 m in all except two years before 2000, the phenomenon of partial mixis developed rather to a rule than an exception since then (Fig. 2a and Supplementary Table S1), with the unique continuous sequence of incomplete turnovers since 2013 mentioned above. Overall, regression analysis for water turnover depth would predict a decrease by 37 m from 1977 to now. In reality, maximal mixing depth for 2016 was with 69 m (Supplementary Fig. S1) even 16 m less than predicted (85 m) and represents the lowest recorded mixing depth since the monitoring started.

The significant long-term decrease of epilimnetic PO_4_-P reflects the re-oligotrophication (Fig. 2a and Supplementary Table S1). Annual maxima mirror the turnover period from January to April, with peaks of >60 µg l^−1^ during the 1980s and 9–20 µg l^−1^ during 2000–2012 (Fig. 2a). The recent strong decrease in epilimnetic PO_4_-P maxima (1.6–2.2 µg l^−1^) co-occurs with decreasing mixing depths during the last four years (inlet in Fig. 2). Also long-term data show a clear correlation between depth of water turnover and vernal epilimnetic phosphorus (Fig. 2b,c and Supplementary Table S2). It was uncertain if the very recent striking PO_4_-P loss in surface waters could be also linked to an ecosystem wide depletion of phosphorus and therefore indicates overdone restoration measures. However, our data show that this phenomenon is clearly linked to lake warming and its strong influence on water turnover dynamics (Fig. 2b,c and Supplementary Table S2). The total PO_4_-P content of the lake (Supplementary Fig. S2) and the hypolimnetic P pool are stable since 1995 (see also^22^) and values are now even increasing (Fig. 2d and Supplementary Table S1) owing to re-solution from anoxic sediments and insufficient export during mixis.

**Figure 3:**
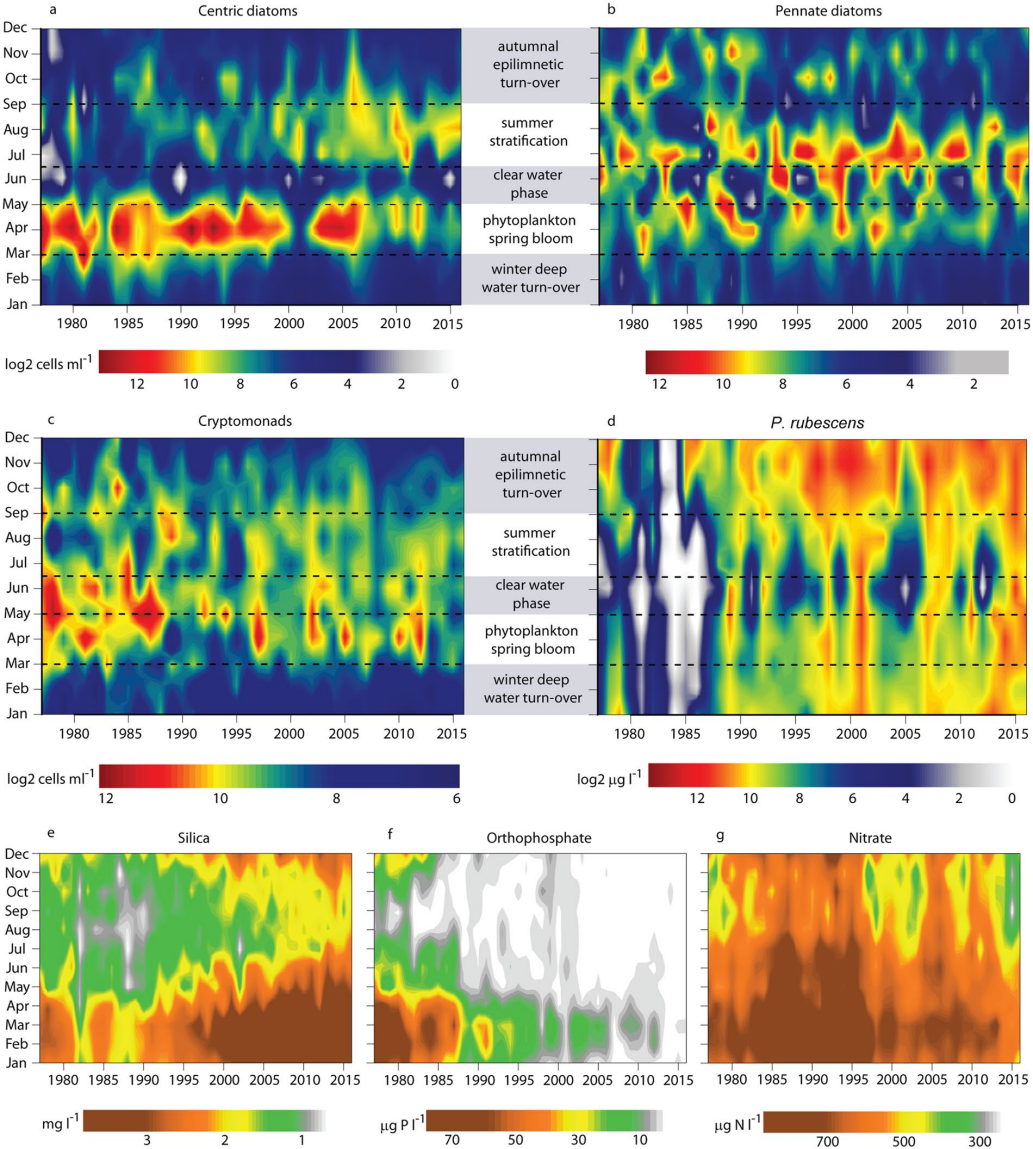
Long-term (1977 to 2016) trends in seasonal successions of abundant algae and their potentially limiting substrates. **(a)** Abundances of centric diatoms, (**b**) pennate diatoms, (**c**) cryptophytes, (**d**) and biomass of *Planktothrix rubescens* (*n* = 480 for all parameters) during characteristic phenological phases (gray-white boxes). Classification of phases followed the terminology of the Plankton Ecology Group (PEG) model published by Sommer *et al*.^15^. Epilimnetic (0–20 m) concentrations of (**e**) silica, (**f**) orthophosphate-P and (**g**) nitrate-N (*n* = 480 for each parameter). All parameters were calculated as depth-volume weighted averages for the water column between 0 and 20 m depth.

After winter, PO_4_-P is the essential growth promotion factor for the phototrophic spring bloom community^15^. Despite the distinct long-term decrease in dissolved P, pronounced vernal mass developments of centric diatoms were recorded until 2000 (Fig. 3a). Notably, centric genera represent the major fraction (average = 79%, median = 86% during the last four decades) of the total diatom assemblage during springtime, whereas pennate diatoms dominate in summer (Fig. 3b and Supplementary Fig. S3a,b). Since 2000, drastic declines of centric diatoms were observed in several years, and spring primary production nearly vanished since 2013 (Supplementary Fig. S3a). Based on the 40 years long-term data we found statistically significant correlations between the parameters: vernal mixis, PO_4_-P, and abundances of centric diatoms (Supplementary Table S2). Centric diatoms, affiliated with the genera *Cyclotella* and *Stephanodiscus*, grow rapidly in spring at low silicium to phosphorus (Si:P) ratios, i.e., algae need relatively high P concentrations^27^. As Si is an essential component for the frustules of diatoms it can be another limiting nutrient apart from P. Typically, silica (SiO_2_) is consumed by centric diatoms during spring and by pennate diatoms thereafter, leading to concentration minima in summer and fall. Epilimnetic maxima are then reached again during vernal turnover. These well-known patterns could also be observed in Lake Zurich during two decades from 1977 on (Fig. 3e). In the last two decades, however, SiO_2_ concentrations were steadily increasing, indicating marginal consumption in spring. Due to the current strong P depletions the Si:P ratio increased during the last decades, thus pennate diatoms (Fig. 3b), which are adapted to high Si:P ratios^15,27^ seemed to be not as much affected by changing nutrient regimes than centric diatoms (Supplementary Fig. S3a,b). Meanwhile SiO_2_ even seemed to accumulate (Supplementary Table S1), and concentrations showed only weak fluctuations during the limnological year, reflecting the decreasing diatom abundances mentioned above. Observable annual SiO_2_ minima might be the result of slightly increasing centric diatom populations in summer (Fig. 3a, Supplementary Table S3 and Fig. S3b). Epilimnetic nitrate values decrease since 1995 (Supplementary Table S1), nevertheless, concentrations are still saturating for autotrophic organisms during spring (Fig. 3g). We found first indications that severe PO_4_-P limitations (Fig. 3f) meanwhile even affected cryptophytes (Fig. 3c and Supplementary Fig. S3c,d), the second dominant group of primary producers during spring. However, we observed only a significant long-term trend for the annual data-sets but not for distinct seasons (Supplementary Table S3). Their major representing genera, *Rhodomonas* and *Cryptomonas*, are typical *r*-strategists^15^, i.e., algae have high growth rates in comparison to competitors, they can develop short lived peaks during the whole year and seem to grow at even minimal P concentrations. Various cryptophyte genera are mixotrophic, i.e. when performing photosynthesis under nutrient depleted conditions, algae may utilize N and P from ingested bacteria as substitutable nutrients^28^. This may explain why long-term data of cryptophytes show much slighter decreases during spring than centric diatoms (Supplementary Fig. S3c). In contrast to the described long-term development of diatoms and cryptophytes, the cyanobacterium *P. rubescens* was strongly favored by the changed ecosystem dynamics during the last four decades (Fig. 3d and Supplementary Tables S2–S3).

**Figure 4:**
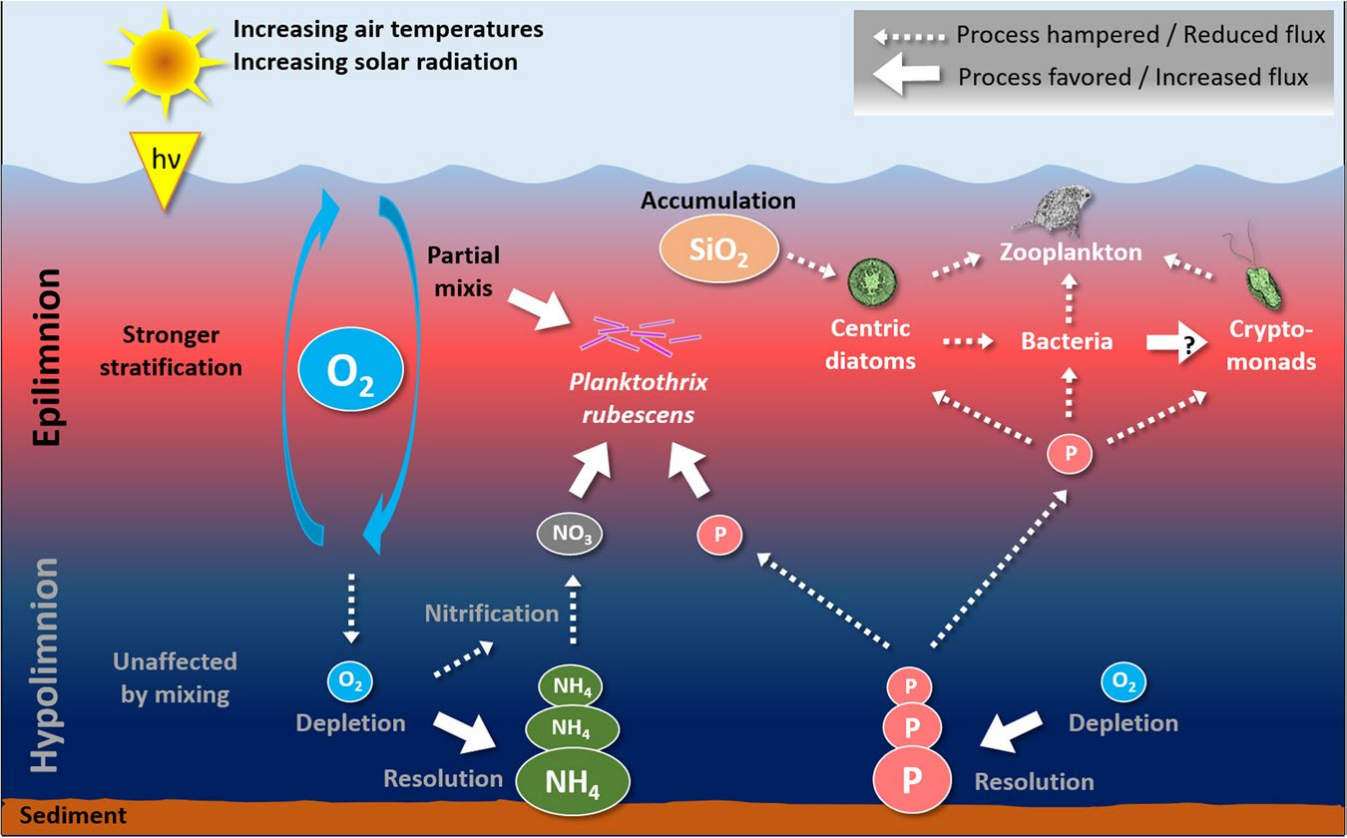
A conceptual view of the major effects of climate warming on water turnover dynamics, primary producers and limiting nutrients in Lake Zurich. Rises in air temperatures cause stronger water column stratifications which impede complete water turnovers (holomixis) during spring. Incomplete mixis favors survival of the cyanobacterium *Planktothrix rubescens*, but reduces the import of limiting nutrients from the hypo- to the epilimnion. The quantitative most important vernal primary producers (centric diatoms and cryptophytes) are negatively affected due to phosphorus limitation. In addition, reduced mixis impedes down-welling of oxygen-rich epilimnetic water, leading to hypolimnetic anoxia and resolution of nutrients from sediments. At present nutrients accumulate in deep water layers owing to insufficient vernal export.

In sum, the reduced primary production of diatoms and cryptophytes may lead to drastic changes of nutrients’ stoichiometry owing to accumulation of otherwise limiting nutrients (e.g. SiO_2_ or Fe) in future (Fig. 4). In addition, incomplete turnovers caused already drastic hypolimnetic oxygen depletions^18,22^, which resulted in changed redox-conditions^23^ and chemical compositions of deep waters (Fig. 4). It is uncertain if the recent drastic declines of vernal algal blooms already propagated along the trophic cascade, i.e. if consumers as Phyllopoda (e.g. daphnids) and copepods suffer from shortage of potential food organisms (Fig. 4). We observed significant negative trends for all metazooplankton entities (Supplementary Table S3 and Fig. S4) and abundances in May (clear-water phase) for the years 2013–2016 are among the lowest values recorded since 1977 (Supplementary Fig. S5). Correlation and multiple regression analyses pointed only to link between cryptophytes and zooplankton but not between the latter and centric diatoms (Supplementary Tables S2 and S4). However, long-term developments also mirror the re-oligotrophication and thus, should be interpreted with caution in regard to the recent reduction of primary production. In addition, regression analyses showed a negative relation between water temperatures and zooplankton, which might hint to direct warming effects on organisms (Supplementary Table S4).

**Figure 5:**
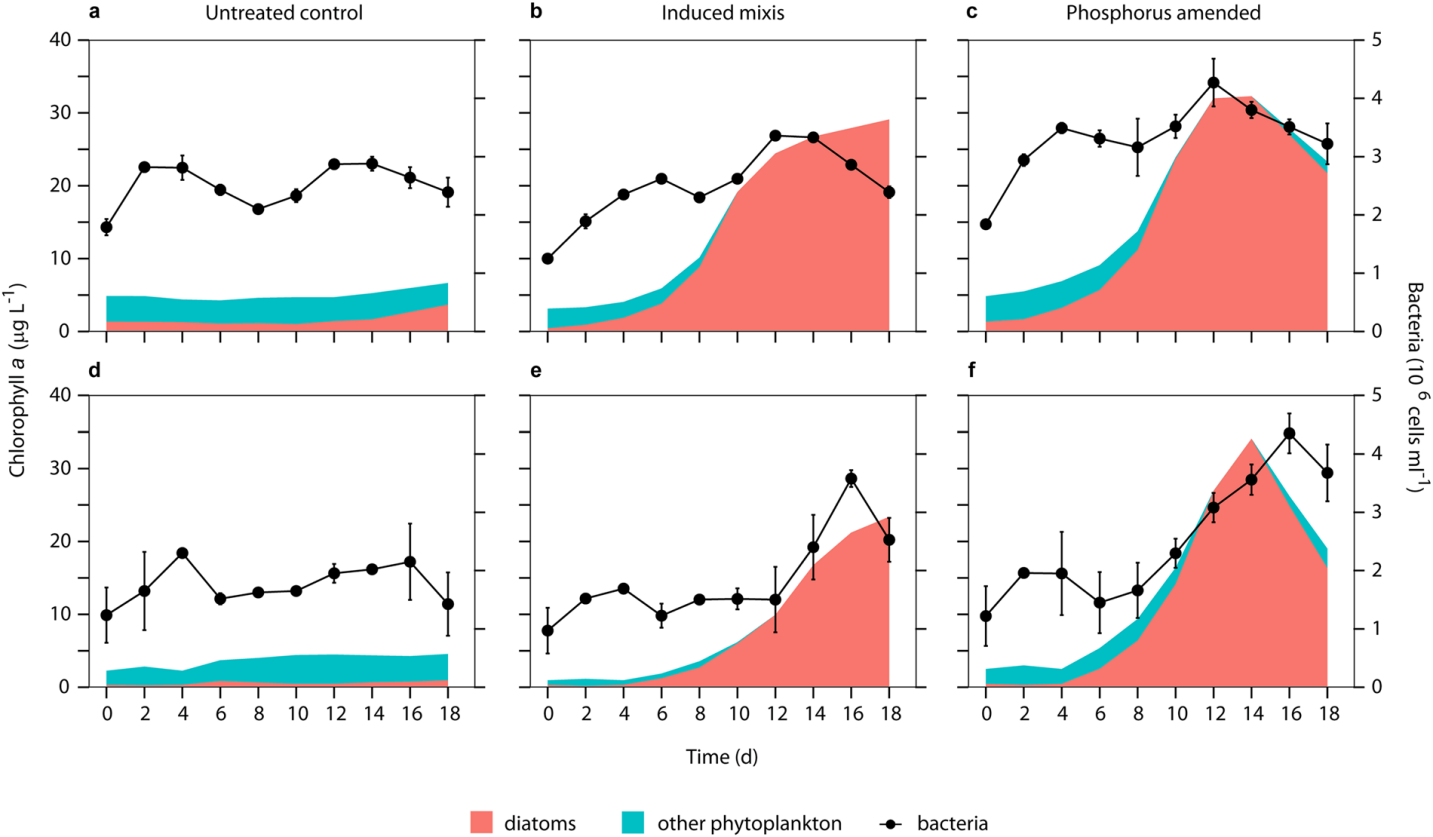
Triggering of diatom blooms via artificial turnover and phosphorus addition. Experiments were conducted during spring periods in 2015 (**a**,**b**,**c**) and 2016 (**d**,**e**,**f**). In untreated (control) surface waters (5 m depth), neither algae nor bacteria showed increased growth (**a**,**d**). Mixing epi- (5 m depth) with hypolimnetic water (100–110 m depth) promoted abundances of diatoms and bacteria (**b**,**e**). A comparable effect was achieved by addition of orthophosphate (40 µg P l^−1^) to epilimnetic (5 m depth) water (**c**,**f**). All treatments were set up as triplicates. Bacterial abundances are shown as averages (*n* = 3) ±one standard deviation.

Lake Zurich is just one of many examples for effective restorations of temperate European lakes^29^. Although this sounds like a successful story of activities by environmental protection agencies and policy, local authorities are now increasingly faced with criticism that nutrient reductions were (too) excessive^30^, causing drastic decreases in lakes’ productivities (e.g., fish stock). Recently, even public debates were initiated about intended re-fertilizations of distinct large European lakes. Restorations aimed in the reduction of autotrophic biomasses to values known from pre-eutrophication periods. Here we show that additional and unexpected drastic decreases in orthophosphate and abundances (of distinct primary producers) are recently observed in a temperate lake. These phenomena are linked to yet over-looked aspects of lake warming, rather than to overdone restoration measures. Two artificial mixis experiments conducted in 2015 and 2016 highlighted the key role of reduced mixis depth in the currently observed productivity loss. They further confirmed the presence of sufficient hypolimnetic nutrients for the promotion of vernal algal blooms. Natural water turnovers reached only to a depth of 95 m in 2015 and 69 m in 2016. In both years, a threefold experimental approach was applied, in which plankton successions were followed for three weeks at light and temperature conditions typically determined during spring periods. (i) In untreated surface waters (5 m depth), neither algal nor bacterial parameters showed any substantial growth (Fig. 5a,d). (ii) Inducing artificial lake turnover by mixing epi- (5 m depth) with hypolimnetic water (100–110 m depth) in a ratio of 1:1, initiated a pronounced increase of chlorophyll *a* concentrations after a few days (Fig. 5b,e). This boost in primary production went in parallel with distinctive bacterial growth (Fig. 5b,e). Bacteria experience considerable growth stimulation after phytoplankton blooms^11,31^ due to raised releases of extracellular dissolved organic carbon (eDOC) by algae. (iii) Epilimnetic water (5 m depth) amended with PO_4_-P (40 µg l^−1^) promoted algal and bacterial growth comparable to the artificial mixis (Fig. 5c,f). Whenever increased algal production was observed, it could be primarily attributed to population dynamics of centric diatoms.

## Conclusion

Lake warming is presently an ongoing process^3,4^ and thus, the probability of serial incomplete water-turnover events and of insufficient hypolimnetic nutrient export will further increase^8^. However, our results indicate that the trophic status of large temperate lakes should not be misinterpreted as to be ultra-oligotrophic. These ecosystems have still the internal capacity to promote distinct phytoplankton blooms given just intensive water (natural or artificial) turnovers again. In consequence, possible intentions to fertilize lakes should be seen very critical by policy makers with respect to sustainable lake management.

## Methods

### Lake Zurich

Lake Zurich refers to Lower Lake Zurich and is a deep (136 m), large (62 km^2^), occasionally holomictic lake situated on the Swiss Plateau^32^. The lake is considered monomictic with a water turn-over in March-April, followed by a period of thermal stratification. Lake Zurich is a source of drinking water for over 1 Million people and of high recreational and economical value for the surrounding urban area. The lake has undergone a long period (1900s until 1970s) of eutrophication due to sewage inputs and runoff from agricultural areas. Thus, concentrations of epilimnetic total phosphorus (TP) reached a final maximum of 120 µg l^−1^ at that time. Since the 1950s, a dual strategy was pursued by constructing sewage treatment plants and reducing nutrient emission from the catchment. Since the 2000s, annual average TP concentrations settled down to 10–15 µg l^−1^. Nitrate (NO_3_-N) concentrations still increased until mid of 1990s, resulting in a high N:P stoichiometry^18^. In parallel with the re-oligotrophication, phototrophic biomass shifted from an algal to a cyanobacterial (*Planktothrix rubescens*) dominated community, owing to changed nutrient stoichiometry and reduced mixing depth^18^. Nevertheless, distinct spring algal blooms were still observed whenever deep mixing took place^13^.

### Long-term data

Recent (2009–2016) depth-profiles of temperature, oxygen, and *P. rubescens* related chlorophyll *a* were measured biweekly (*n* = 192) near the deepest point of the lake using the multi-parameter probes YSI 6600 (YSI Incorp., Yellow Springs, OH, USA) and TS-16–12 fluoroprobe (bbe Moldaenke GmbH, Kronshagen, Germany). Parameters were measured in 1 m intervals from 0–120 m. Phosphorus (total and particulate) and orthophosphate concentrations were determined monthly for 17 distinct sampling depths from 0–136 m.

In course of an ongoing long-term monitoring program since 1977, samples are collected by the Zurich Water Supply near the deepest point of the lake. Physico-chemical parameters (nitrate, oxygen, phosphorus, silica, and water temperature) were analyzed monthly for 19 distinct depths from 0–136 m (*n* = 9,120). Biotic parameters (abundances of diatoms and cryptophytes, biomass of *P. rubescens*) were analyzed monthly for 14 distinct depths from 0–136 m (*n* = 6,720). The quantification of algae is based on microscopic evaluation of fixed samples^33^. Epilimnetic values were calculated from depth-weighted averages of samples (depths: 0, 1, 2.5, 5, 7.5, 10, 12.5, 15 and 20 m) between 0–20 m. Hypolimnetic values were calculated from depth-weighted averages of samples (depths: 100, 110, 120, 130, 136 m) between 100–136 m. We used the 6 mg O_2_ l^−1^ isoline as a proxy for the vernal mixing depth^18,21^. The quantification of zooplankton groups is based on microscopic evaluation of formaldehyde fixed samples (net-hauls from 0–20 m, and 20–136 m). Plankton nets in twin arrangement with tilt-closing mechanisms were applied^34^. The relatively small mesh-size of 95 µm should guarantee that even nauplius larvae are collected in a quantitative way^34^.

### Experimental setup

Experiments were run in March 2015 and March 2016 and lasted for 18 days each. All treatments were conducted in triplicates and waters were incubated in 5 l bottles at 10 °C and 40 µmol m^−2^ s^−1^ light intensity, and at 12:12 h day: night cycle. Water for the three different treatments (see below) was taken from 5 m and 100 (110) m near to the deepest point of Lake Zurich (N 47°17.147′, E 8°35.460′) and filtered through a 100 µm filter to exclude larger predators. The control treatments consisted of 4 l untreated water from 5 m depth with initial TP concentrations of 4 µg P l^−1^ in 2015 and 11 µg P l^−1^ in 2016. Artificial turnover treatments consisted of 2 l surface water (5 m) mixed with 2 l deep water (from 110 m in 2015, and from 100 m in 2016; these depths corresponded to the deepest still aerobic zones in Lake Zurich). Mixing with deep waters resulted in TP concentrations of 22 µg P l^−1^ in 2015 and 35 µg P l^−1^ in 2016. For the phosphorus amended treatments we added K_2_HPO_4_ to a final concentration of 40 µg P l^−1^ to 4 l of surface water (5 m). Thus, the initial measured TP concentrations in these treatments were 40 µg P l^−1^ in 2015 and 50 µg P l^−1^ in 2016. Measurements of chlorophyll *a* and bacterial abundance were made every second day. Chlorophyll *a* was measured in a 25 ml cuvette with the TS-16–12 fluoroprobe (see above). For the quantification of bacteria, 10 ml subsamples were fixed with 0.5 ml formaldehyde (2% end concentration), stained with SYBR-Green and analyzed with an InFlux V-GS Flow Cytometer (Cytopeia Inc.).

### Statistical data analyses

All statistical analyses were performed using R. We analyzed long-term (40 years) data for significant trends by Mann Kendall trend tests. To account for effects of significant residual autocorrelations, we applied the “trend-free pre-whitening” procedure^35^ incorporated in the package “zyp” to datasets (annual mixing depth, maximum *P. rubescens* biomass, as well as maximum diatom and cryptophytes abundances in spring and summer). Seasonal datasets (orthophosphate, nitrate, silica, centric diatoms, cryptophytes, *P. rubescens* and zooplankton groups) were subjected to seasonal MK trend test with integrated serial dependence removal^36^ by using the package “EnvStats”. The change point detection in whole-lake orthophosphate content was estimated by using two-segment piecewise linear regressions after Crawley (2012)^37^. First, a wide range around the assumed change point was chosen visually. Second, residual standard errors were calculated by running two-segment linear regressions for each possible change point (iterative searching). Finally, the change point was estimated as the model with lowest residual standard error. Spearman’s correlation analyses were carried out on detrended (achieved by differencing) and not detrended data to test for correlations between mixing depth, concentrations of major chemical parameters and abundances of organisms, as well as *P. rubescens* biomass. To study the effect of water temperature, orthophosphate and zooplankton on centric diatoms, as well as of water temperature, centric diatoms and cryptophytes on zooplankton, multiple linear regressions were applied. No significant deviations from normality or autocorrelations of the models residuals were found.

### Data availability

The authors declare that data supporting the findings of this study are available from the authors upon request.

## Electronic supplementary material

Supplementary Information
