# Supplementary material for: Abrupt stop of deep water turnover with lake warming: Drastic consequences for algal primary producers
Source: Sci Rep. 2017 Oct 23;7:13770. doi: 10.1038/s41598-017-13159-9 (PMC5653828; doi:10.1038/s41598-017-13159-9)
Supplement: Supplementary file 1 — Supplementary Information [file 41598_2017_13159_MOESM1_ESM.pdf]

Abrupt stop of deep water turnover with lake warming: Drastic consequences for algal primary producers

Yana Yankova[1], Stefan Neuenschwander[1], Oliver Köster[2], and Thomas Posch[1]*

[1]Limnological Station, Department of Plant and Microbial Biology, University of Zurich, Seestrasse 187, CH-8802 Kilchberg, Switzerland,

[2]Zurich Water Supply, Hardhof 9, CH-8021 Zurich, Switzerland.

*posch@limnol.uzh.ch

Supplementary Information

Supplementary Figures & Legends S1 – S5 and Tables S1 – S4

*Corresponding author:

PD Dr. Thomas Posch

Limnological Station, Department of Plant and Microbial Biology, University of Zurich

Seestrasse 187, CH-8802 Kilchberg, Switzerland

Phone: 0041 44 634 9224

Fax: 0041 44 634 9225

e-mail: posch@limnol.uzh.ch

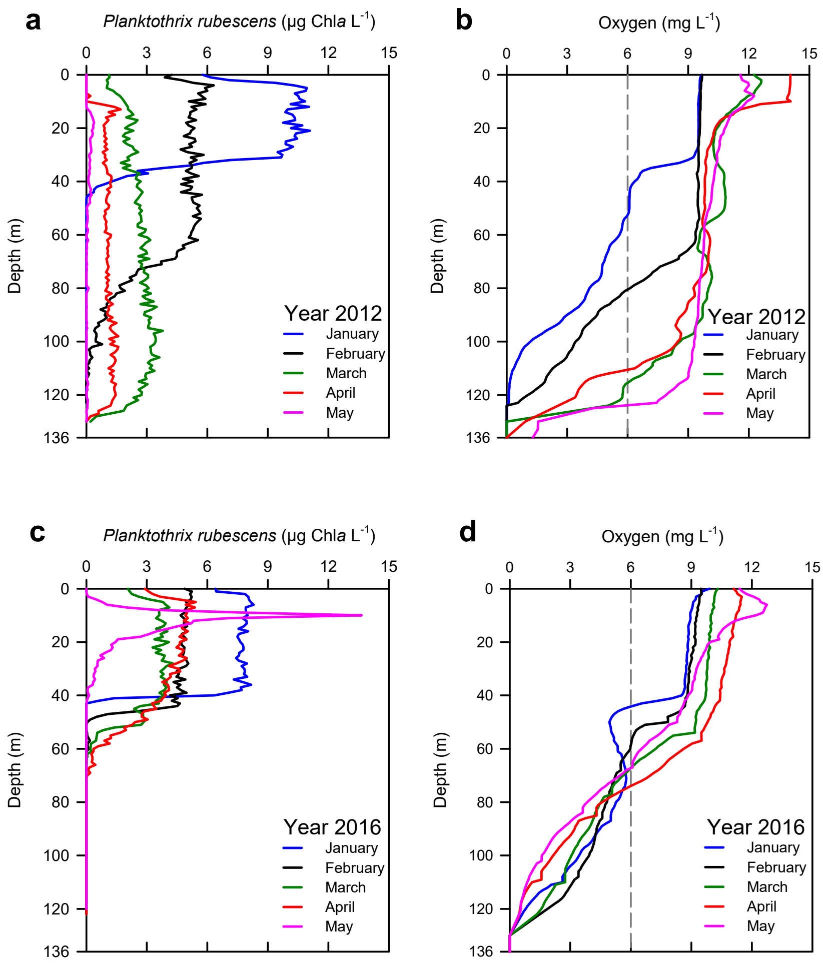

Supplementary Figure S1 | Monthly vertical profiles (January to May) of the cyanobacterium *Planktothrix rubescens* and oxygen concentrations in two years with different water turnover dynamics. In the year 2012 vernal water turnover (mixis depth) affected water strata down to a depth of ~120m, resulting in a deep entrainment of *P. rubescens* (**a**) and a strong oxygen enrichment of the hypolimnion (**b**). Note that the proxy for mixis intensity ($\geq$6 mg O_2 L^{-1}; gray stippled line in (**b**) and (**d**)) adequately reflected the depth of water turnover. Such deep mixis events led to a drastic decline of *P. rubescens* (due to the collapse of their gas vesicles), causing a minimal starting population in May. In the year 2016 vernal water turnover affected water strata only down to ~70m. This partial mixis was reflected by the vertical distribution of *P. rubescens* (**c**) and oxygen concentrations (**d**). Due to the partial mixis a large proportion of *P. rubescens* survived, which formed a densely stratified starting population already in May.

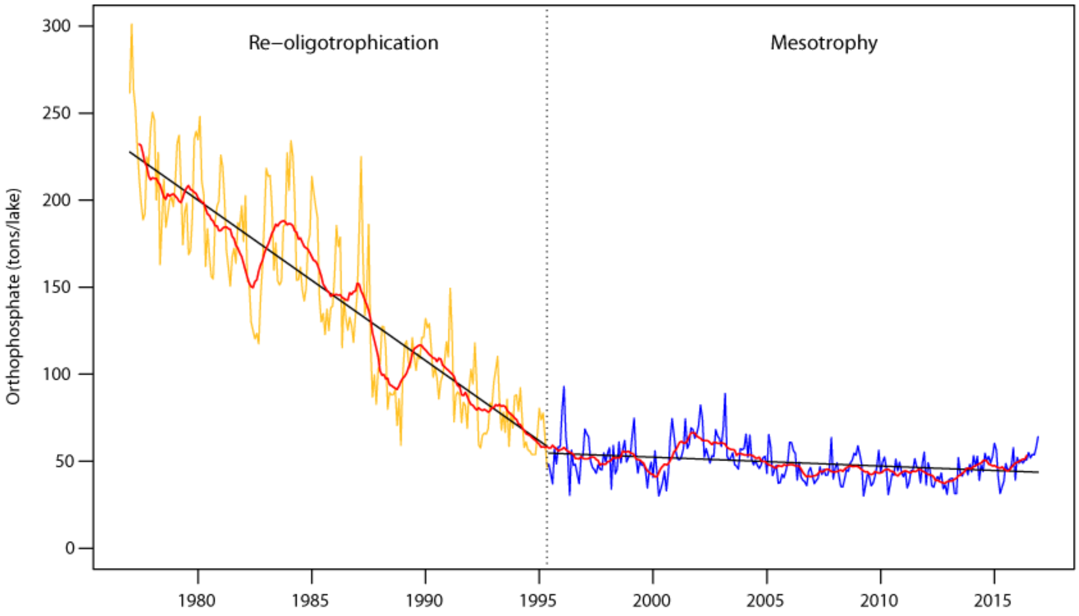

Supplementary Figure S2 | Change point in the development of total orthophosphate content. The change point (dotted line) in whole-lake orthophosphate content (n = 480) was determined for May 1995 by applying an iterative searching approach (details in Methods). Piece-wise regressions (black solid lines, $f = b*x + a$) signify transition from a re-oligotrophication mediated rapid orthophosphate decrease (orange line) to a stable oligo-mesotrophic status (blue line). Red line: continuous running average (n = 12).

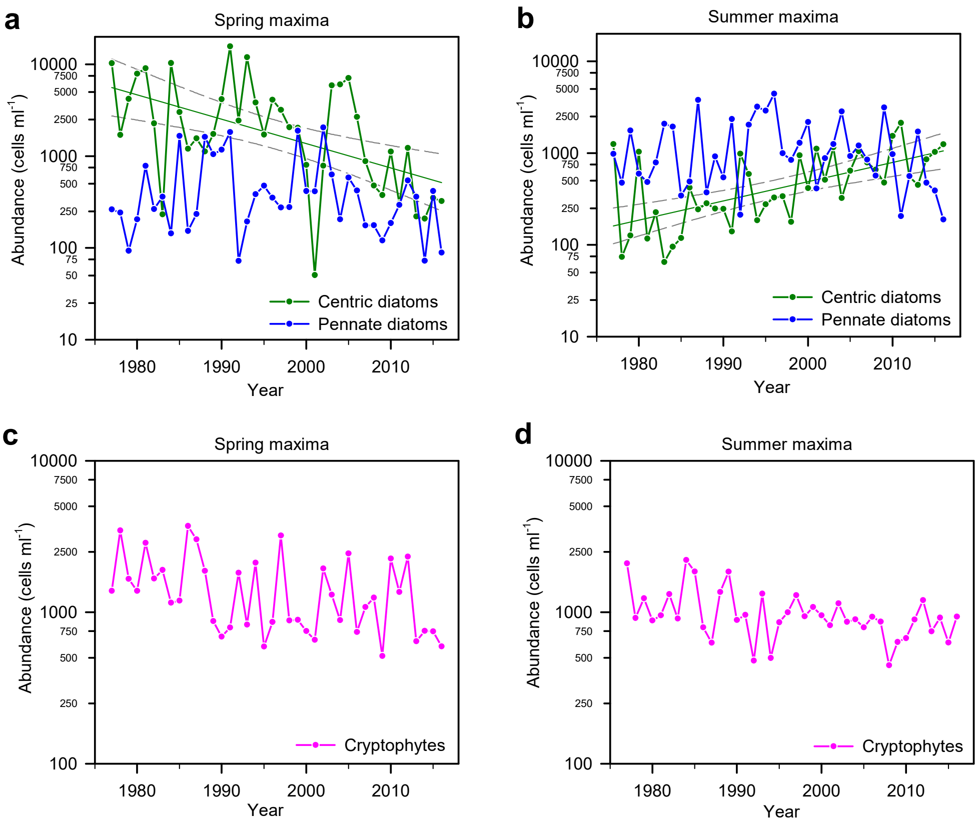

Supplementary Figure S3 | Seasonal trends of centric / pennate diatoms and cryptophytes abundances between 1977 and 2016. a,**b,** Maximal abundances of centric and pennate diatoms in spring (**a**) and summer (**b**). Linear regressions (solid lines with confidence intervals; log(y) = $b*x + a$, $r^2 = 0.29$ for centric diatoms in spring, $r^2 = 0.39$ for centric diatoms in summer) were included when significant time trends on detrended data (see Supplementary Table S3) were detected. Maximal abundances of cryptophytes in spring (**c**) and summer (**d**). No significant time trends were detected for detrended data about cryptophytes (see Supplementary Table S3). Spring = March-May, summer = July-October. For all parameters: $n = 40$.

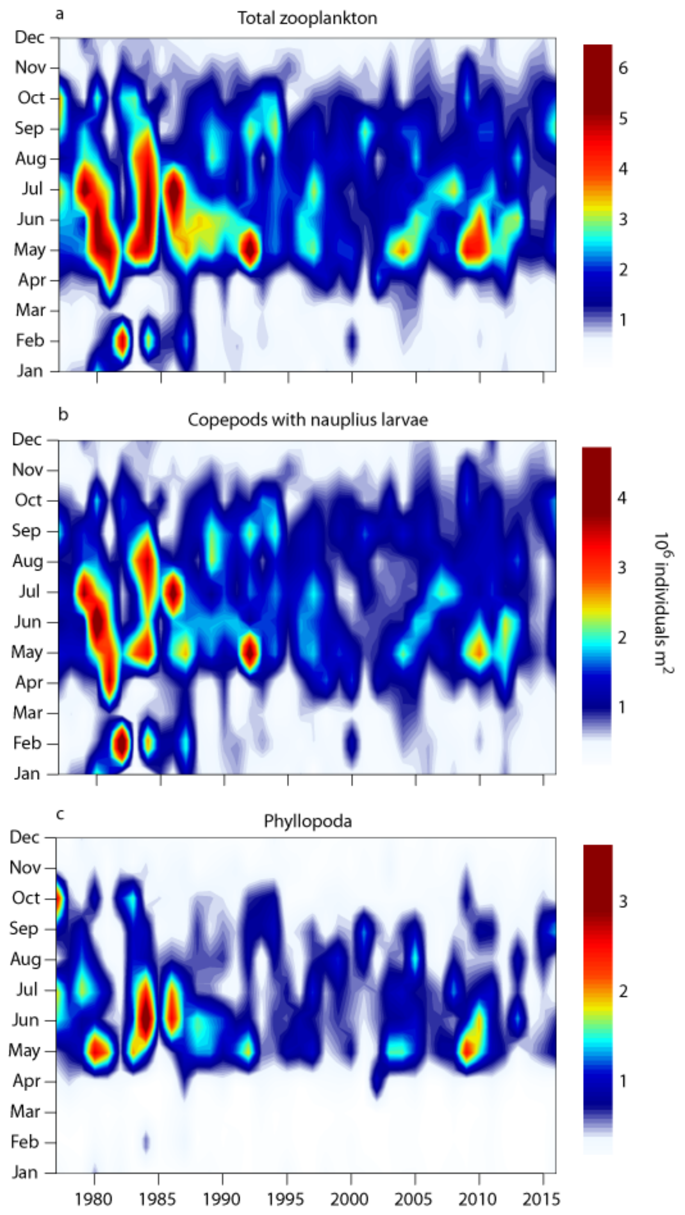

Supplementary Figure S4 | Zooplankton abundance in Lake Zurich for the period 1977-2016. Monthly abundance (n = 480 each) of total zooplankton (**a,** copepods + nauplius larvae + Phyllopoda), copepods with nauplius larvae (**b**) and Phyllopoda (**c**), averaged for 0-136 m.

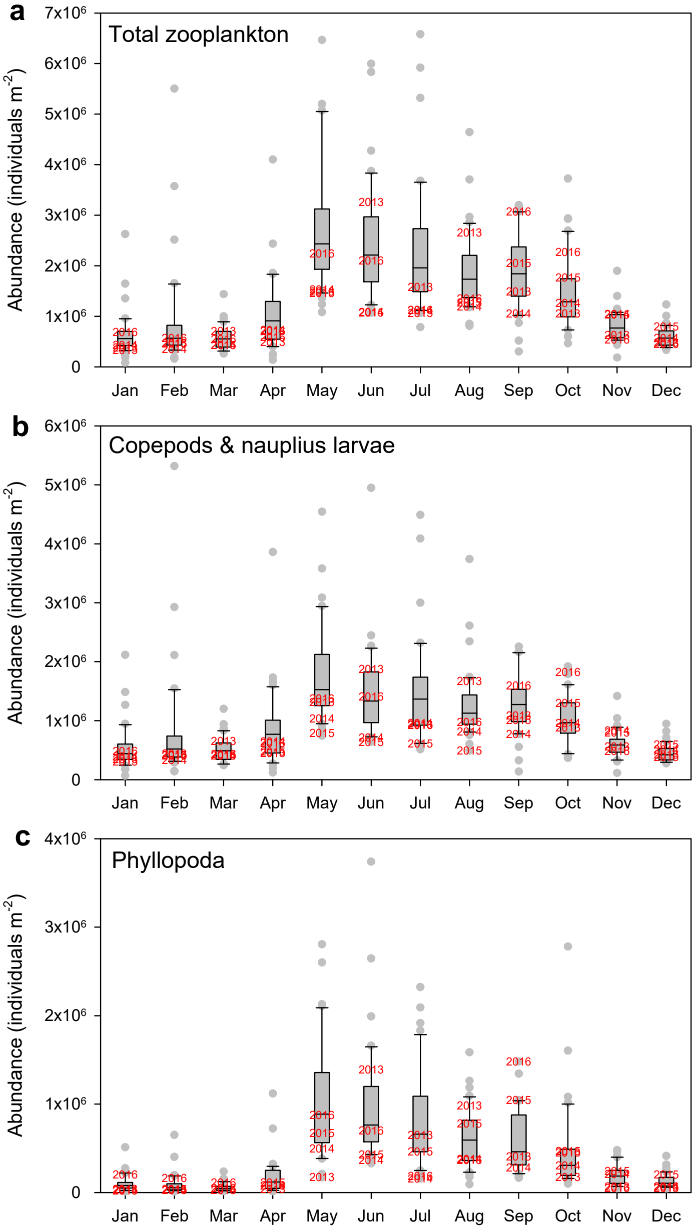

Supplementary Figure S5 | Seasonality pattern of zooplankton abundance in Lake Zurich for the period 1977-2016. Monthly abundance of total zooplankton (**a**, copepods + nauplius larvae + Phyllopoda), copepods with nauplius larvae (**b**) and Phyllopoda (**c**), averaged for 0-136 m. The bars show the 25th, 50th and 75th percentiles, whiskers stand for the 10th and the 90th percentiles and grey points show outliers. Values for the four last years (2013-2016) are shown in red with years as labels. For the 40 years period, the highest zooplankton abundances were reached in May (clear water phase of Lake Zurich). For all parameters: n = 480.

Supplementary Table S1 | Statistical time-trends of physico-chemical long-term data (1977 until 2016) of Lake Zurich. Trends were analyzed for the whole datasets and for periods before and after 1995. This workflow was chosen as orthophosphate values showed a change point in 1995. Phosphorus concentrations in lakes are major parameters to characterize the trophic status.

Parameter	n	Seasonal Mann Kendall's tau (period = 12)	Mann Kendall's tau	Autocorrelation of detrended time series	p-value	Significant trend
**Epilimnetic orthophosphate**						
whole period	480	-0.520	-	-	< 0.001	negative
< 1995	216	-0.569	-	-	< 0.001	negative
≥ 1995	264	-0.212	-	-	0.018	negative
**Hypolimnetic orthophosphate**						
whole period	480	-0.443	-	-	< 0.001	negative
< 1995	216	-0.484	-	-	< 0.001	negative
≥ 1995	264	0.204	-	-	0.026	positive
**Epilimnetic silica**						
whole period	480	0.541	-	-	< 0.001	positive
< 1995	216	-0.158	-	-	0.212	no trend
≥ 1995	264	0.470	-	-	< 0.001	positive
**Epilimnetic nitrate**						
whole period	480	-0.264	-	-	0.004	negative
< 1995	216	0.510	-	-	< 0.001	positive
≥ 1995	264	-0.350	-	-	0.004	negative
**Average mixing depth**	40	-	-0.446	0.004	< 0.001	negative

Supplementary Table S2 | Spearman's correlation analysis of interactions between detrended and not detrended average mixing depth, key biological and chemical variables for the period 1977-2016 (all *n* = 40). All variables (except mixis depth) are maxima of weighted averages for 0-20 m, except zooplankton (for 0-136 m). Zooplankton = copepods with nauplius larvae + Phyllopoda. Significant correlations are bold.

	Spearman's *rho* (detrended)	*p*-value (detrended)	Spearman's *rho* (not detrended)	*p*-value (not detrended)
Mixis depth – PO_4-P $_{jan\text{-}mar}$	**0.631**	**< 0.001**	**0.713**	**< 0.001**
Mixis depth – NO_3-N $_{jan\text{-}mar}$	**0.443**	**0.005**	**0.510**	**0.001**
Mixis depth – SiO_2 $_{jan\text{-}mar}$	**0.390**	**0.014**	**-0.411**	**0.008**
Mixis depth – centric diatoms $_{mar\text{-}may}$	0.232	0.156	**0.472**	**0.002**
Mixis depth – cryptomonads $_{mar\text{-}may}$	0.225	0.169	0.278	0.083
Mixis depth – zooplankton $_{apr\text{-}jun}$	0.129	0.434	0.165	0.310
Mixis depth – *P. rubescens* $_{mar\text{-}may}$	**-0.716**	**< 0.001**	**-0.701**	**< 0.001**
PO_4-P $_{jan\text{-}mar}$ – NO_3-N $_{jan\text{-}mar}$	**0.518**	**0.001**	**0.467**	**0.002**
PO_4-P $_{jan\text{-}mar}$ – SiO_2 $_{jan\text{-}mar}$	**0.399**	**0.012**	**-0.733**	**< 0.001**
PO_4-P $_{jan\text{-}mar}$ – centric diatoms $_{mar\text{-}may}$	**0.645**	**< 0.001**	**0.616**	**< 0.001**
PO_4-P $_{jan\text{-}mar}$ – cryptomonads $_{mar\text{-}may}$	0.188	0.252	**0.395**	**0.012**
PO_4-P $_{jan\text{-}mar}$ – zooplankton $_{apr\text{-}jun}$	0.165	0.317	**0.379**	**0.016**
PO_4-P $_{jan\text{-}mar}$ – *P. rubescens* $_{mar\text{-}may}$	**-0.659**	**< 0.001**	**-0.840**	**< 0.001**
NO_3-N $_{jan\text{-}mar}$ – SiO_2 $_{jan\text{-}mar}$	0.221	0.177	**-0.622**	**< 0.001**
NO_3-N $_{jan\text{-}mar}$ – centric diatoms $_{mar\text{-}may}$	**0.372**	**0.020**	**0.468**	**0.002**
NO_3-N $_{jan\text{-}mar}$ – cryptomonads $_{mar\text{-}may}$	0.079	0.634	0.169	0.295
NO_3-N $_{jan\text{-}mar}$ – zooplankton $_{apr\text{-}jun}$	0.151	0.357	0.261	0.104
NO_3-N $_{jan\text{-}mar}$ – *P. rubescens* $_{mar\text{-}may}$	**-0.428**	**0.007**	**-0.412**	**0.008**
SiO_2 $_{jan\text{-}mar}$ – centric diatoms $_{mar\text{-}may}$	0.259	0.112	**-0.374**	**0.017**
SiO_2 $_{jan\text{-}mar}$ – cryptomonads $_{mar\text{-}may}$	-0.005	0.975	-0.272	0.089
SiO_2 $_{jan\text{-}mar}$ – zooplankton $_{apr\text{-}jun}$	0.043	0.793	-0.281	0.079
SiO_2 $_{jan\text{-}mar}$ – *P. rubescens* $_{mar\text{-}may}$	-0.295	0.069	**0.569**	**0.001**
Centric diatoms $_{mar\text{-}may}$ – cryptomonads $_{mar\text{-}may}$	0.118	0.473	0.191	0.237
Centric diatoms $_{mar\text{-}may}$ – zooplankton $_{apr\text{-}jun}$	0.076	0.646	0.255	0.113
Centric diatoms $_{mar\text{-}may}$ – *P. rubescens* $_{mar\text{-}may}$	**-0.385**	**0.016**	**-0.566**	**< 0.001**
Cryptomonads $_{mar\text{-}may}$ – zooplankton $_{apr\text{-}jun}$	0.245	0.132	**0.440**	**0.005**
Cryptomonads $_{mar\text{-}may}$ – *P. rubescens* $_{mar\text{-}may}$	-0.226	0.167	**-0.427**	**0.006**
Zooplankton $_{apr\text{-}jun}$ – *P. rubescens* $_{mar\text{-}may}$	-0.250	0.125	**-0.431**	**0.005**

Supplementary Table S3 | Statistical time-trends of biological long-term data (1977 until 2016) of Lake Zurich. Trends were analyzed for the whole datasets and for periods before and after 1995 (for centric and pennate diatoms, cryptophytes and *Planktothrix rubescens*). This workflow was chosen as orthophosphate values showed a change point in 1995. Phosphorus concentrations in lakes are major parameters to characterize the trophic status.

Parameter	n	Seasonal Mann Kendall's tau (period = 12)	Mann Kendall's tau	Autocorrelation of detrended time series	p-value	Significant trend
**Centric diatoms**						
all seasons	480	-0.026	-	-	0.594	no trend
spring	40	-	-0.357	0.047	0.001	negative
< 1995	18	-	0.176	-0.153	0.343	no trend
≥ 1995	22	-	-0.400	0.581	0.012	negative
summer	40	-	0.498	-0.051	< 0.001	positive
**Pennate diatoms**						
all seasons	480	-0.106	-	-	0.004	negative
spring	40	-	-0.069	0.114	0.545	no trend
< 1995	18	-	0.088	0.130	0.650	no trend
≥ 1995	22	-	-0.257	-0.150	0.110	no trend
summer	40	-	0.009	-0.121	0.942	no trend
**Cryptophytes**						
all seasons	480	-0.127	-	-	0.010	negative
spring	40	-	-0.217	-0.011	0.052	no trend
< 1995	18	-	-0.117	0.223	0.536	no trend
≥ 1995	22	-	-0.123	-0.203	0.450	no trend
summer	40	-	-0.217	0.004	0.053	no trend
P. rubescens						
all seasons	480	0.442	-	-	< 0.001	positive
spring	40	-	0.527	0.057	< 0.001	positive
< 1995	18	-	0.412	0.122	0.023	positive
≥ 1995	22	-	0.209	0.025	0.194	no trend
summer	40	-	0.320	0.190	0.004	positive
**Zooplankton**						
total	480	-0.138	-	-	0.001	negative
Copepods and nauplius larvae	480	-0.092	-	-	0.029	negative
Phyllopoda	480	-0.176	-	-	< 0.001	negative

Centric and pennate diatoms/cryptophytes/*P. rubescens* spring = maximal abundance between March and May. Centric and pennate diatoms/cryptophytes/*P. rubescens* summer = maximal abundance between July and October. Zooplankton total = copepods with nauplius larvae + Phyllopoda.

Supplementary Table S4 | Summary of multiple regression analyses for variables predicting (a) centric diatoms and (b) zooplankton for the period 1977-2016 (all n = 40). All biological variables and PO_4-P are maxima of weighted averages for 0-20 m, except zooplankton (for 0-136 m). Temperatures are mean values of weighted averages for 0-20 m. Zooplankton = copepods with nauplius larvae + Phyllopoda. Significant p-values are bold.

(a)

	Centric diatoms $_{mar-may}$		
Variable	β	*t*	*p*
Temperature $_{mar-may}$	0.205	1.213	0.233
PO_4-P $_{jan-mar}$	0.554	3.255	**0.002**
Zooplankton $_{apr-jun}$	0.094	0.601	0.551
R^2	0.267		
F-statistic	4.36 (df = 3;36), ***p* = 0.010**		

(b)

	Zooplankton $_{apr-jun}$		
Variable	β	*t*	*p*
Temperature $_{apr-jun}$	-0.305	-2.121	**0.041**
Cryptomonads $_{mar-may}$	0.331	2.311	**0.027**
Centric diatoms $_{mar-may}$	0.198	1.378	0.177
R^2	0.262		
F-statistic	4.26 (df = 3;36), ***p* = 0.011**		
